# Assessment of Runoff and Sediment Yields Using the AnnAGNPS Model in a Three-Gorge Watershed of China

**DOI:** 10.3390/ijerph9051887

**Published:** 2012-05-16

**Authors:** Lizhong Hua, Xiubin He, Yongping Yuan, Hongwei Nan

**Affiliations:** 1 Department of Spatial Information Science and Engineering, Xiamen University of Technology, Xiamen 361024, China; Email: lzhua@xmut.edu.cn; 2 Institute of Mountain Hazards and Environment, Chinese Academy of Sciences, Chengdu 610041, China; Email: nanhw@126.com; 3 USEPA-Office of Research and Development, 944 E. Harmon Ave, Las Vegas, NV 89119, USA

**Keywords:** AnnAGNPS, modeling, Daning River watershed, Three-Gorge, erosion and sediment

## Abstract

Soil erosion has been recognized as one of the major threats to our environment and water quality worldwide, especially in China. To mitigate nonpoint source water quality problems caused by soil erosion, best management practices (BMPs) and/or conservation programs have been adopted. Watershed models, such as the Annualized Agricultural Non-Point Source Pollutant Loading model (AnnAGNPS), have been developed to aid in the evaluation of watershed response to watershed management practices. The model has been applied worldwide and proven to be a very effective tool in identifying the critical areas which had serious erosion, and in aiding in decision-making processes for adopting BMPs and/or conservation programs so that cost/benefit can be maximized and non-point source pollution control can be achieved in the most efficient way. The main goal of this study was to assess the characteristics of soil erosion, sediment and sediment delivery of a watershed so that effective conservation measures can be implemented. To achieve the overall objective of this study, all necessary data for the 4,184 km^2^ Daning River watershed in the Three-Gorge region of the Yangtze River of China were assembled. The model was calibrated using observed monthly runoff from 1998 to 1999 (Nash-Sutcliffe coefficient of efficiency of 0.94 and *R*^2^ of 0.94) and validated using the observed monthly runoff from 2003 to 2005 (Nash-Sutcliffe coefficient of efficiency of 0.93 and *R*^2^ of 0.93). Additionally, the model was validated using annual average sediment of 2000–2002 (relative error of −0.34) and 2003–2004 (relative error of 0.18) at Wuxi station. Post validation simulation showed that approximately 48% of the watershed was under the soil loss tolerance released by the Ministry of Water Resources of China (500 t·km^−2^·y^−1^). However, 8% of the watershed had soil erosion of exceeding 5,000 t·km^−2^·y^−1^. Sloping areas and low coverage areas are the main source of soil loss in the watershed.

## 1. Introduction

Soil erosion has increased throughout the 20th century [1], and has become an extremely serious environmental problem worldwide. It has been recognized as a threat to the productivity of the farms and the quality of surface waters in the Three-Gorge area of the Yangtze River of China. The region is subjected to flooding, soil erosion and sedimentation hazards leading to environmental, social and economic problems. However, there is only limited research available to describe the erosion, sedimentation, and water quality dynamics on a watershed scale in this region. Thus, the accurate quantification of soil erosion and sediment in the watersheds of the region is urgently needed and essential for efficiently planning land use, enhancing agricultural production and productivity, reducing reservoir sedimentation and improving stream water quality. 

Watershed models are considered as a cost-effective and time-efficient method for assessment of pollutant loads and simulation of watershed processes and management practices in an effort to address non-point source pollution [2]. Several watershed-scale hydrological and water quality models, such as AnnAGNPS (Annualized AGricultural Non-Point Source) [3] and SWAT (Soil and Water Assessment Tool) [4], have been developed over the past decades to evaluate the hydrologic and water quality responses of a watershed to alternative management practices. AnnAGNPS, a parameter distributed and semi-physically based model, is designed to simulate water, sediment and chemical movement from agricultural watersheds on a continuous daily time step [5]. AnnAGNPS was developed as an expansion of the capabilities of the single event AGNPS with improved technology and significantly advanced features. The single event AGNPS model has received extensive evaluation and validation in the United States [6,7,8], Canada [9], Italy [10], and Germany [11]. 

AnnAGNPS has been successfully used for hydrology, sediment and nutrient loading predictions and evaluation of cost-effective alternative policy scenarios over a wide range of environments in the United States [12,13,14,15,16,17], Czechoslovakia [18], Nepal [19], Australia [2], Malaysia [20], and India [21]. 

The objectives of this study were to: (1) calibrate and validate the capability of AnnAGNPS to predict runoff on the watershed using field observed data; and (2) evaluate the characteristics of soil erosion, sediment and sediment delivery on the Daning River watershed after AnnAGNPS is validated.

## 2. Results and Discussion

**Figure 1:**
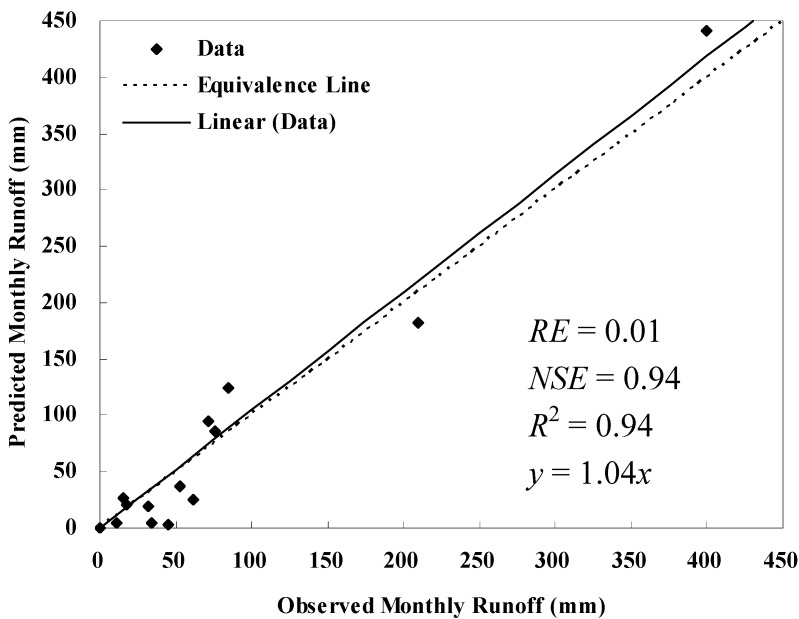
Comparison of observed and predicted monthly runoff at Wuxi hydrological station during calibration year 1998–1999.

**Figure 2:**
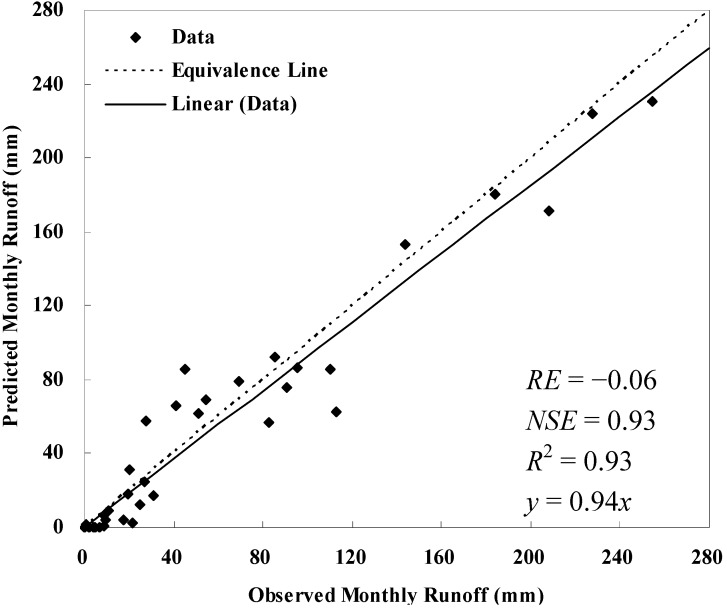
Comparison of observed and predicted monthly runoff at the Wuxi hydrological station during validation year 2003–2005.

**Figure 3:**
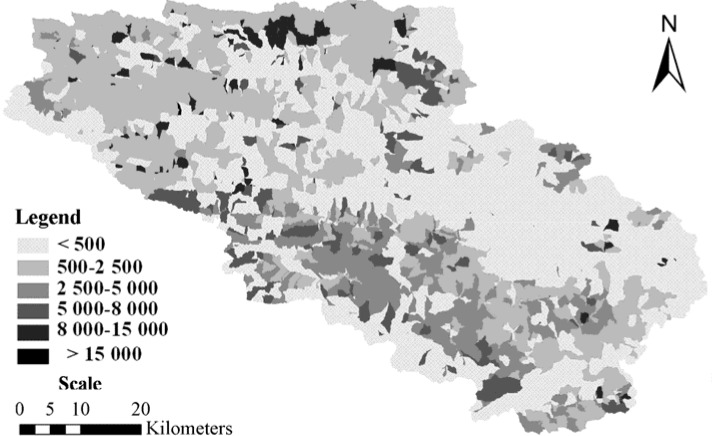
Spatial distribution map of average annual soil erosion by AnnAGNPS in the Daning River watershed.

**Figure 4:**
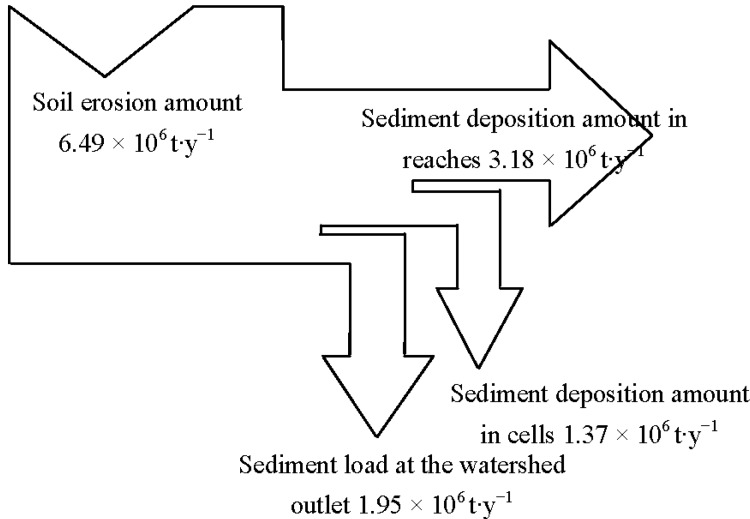
Annual sediment budget for Daning River watershed predicted by AnnAGNPS. Arrow thickness is proportional to the amount of sediment input or output.

**Figure 5:**
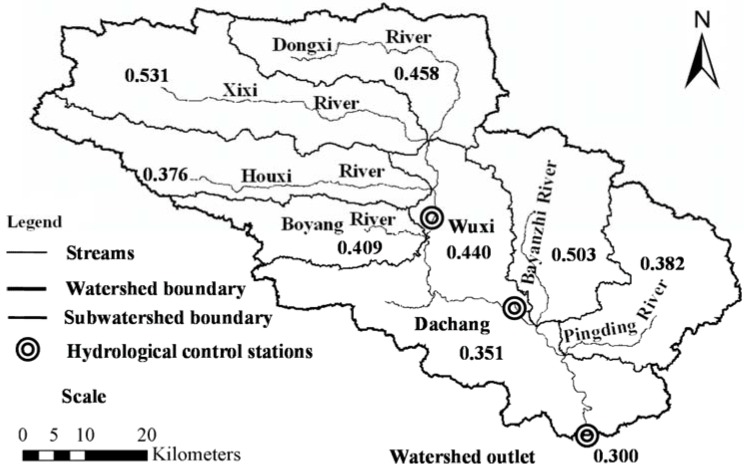
Spatial distribution of sediment delivery ration (SDR) predicted by AnnAGNPS in the Daning River watershed.

Calibration and validation results for runoff are shown in Table 1. AnnAGNPS simulated and field observed monthly runoff from precipitation events which were available for model evaluation at the Wuxi gauging station are displayed in Figure 1 and Figure 2. Characteristics of soil erosion and sediment yield within Daning River watershed for the current condition simulation are given in Table 2, Table 3, Table 4 and Figure 3, Figure 4. Results for sediment delivery ratio are given in Figure 5.

**Table 1:** Statistics concerning the AnnAGNPS simulations of monthly runoff at Wuxi hydrological station during calibration and validation period.

Phase	Values	Mean (mm)	SD ^a^ (mm)	RE ^b^	*R*^2 c^	k	NSE ^d^	RMSE(%) ^e^
Calibration	Observed	81.97	109.33					
	Simulated	82.05	120.96	0.01	0.94	1.04	0.94	28
Validation	Observed	61.72	69.62					
	Simulated	58.08	65.84	−0.06	0.93	0.94	0.93	29

^a^ Standard deviation; ^b^ Relative error; ^c^ Coefficient of determination; ^d^ Nash-Sutcliffe coefficient of efficiency; ^e^ Root mean square error.

### 2.1. Model Evaluation

As shown in Table 1, the total runoff predicted by AnnAGNPS compared well with the observed data (*RE* = 0.01) during the calibration period (1998–1999). 

In addition, monthly runoff predicted by AnnAGNPS also compared well with the observed data (*NSE* = 0.94), and the regression of the monthly predicted runoff with the observed runoff on the line of equal values was satisfactory, with an *R*-square value of 0.94 (*p* < 0.05) and a slope of 1.04. The mean and standard deviation of simulated runoff were close to the corresponding observed runoff as shown in Table 1. Thus, the model can predict monthly runoff well after calibration (Figure 1). 

Comparisons of predicted and observed total runoff during the validation period (2003–2005) produced a *RE* of −0.06; and comparisons of predicted and observed monthly runoff during validation period produced a *NSE* of 0.93 and an R-square value of 0.93 (*p* < 0.05), which again demonstrates a satisfactory fit with field data (Figure 2). 

Studies performed by Licciardello *et al*. [22] in a Mediterranean watershed produced a *NSE* of 0.76 and *RE* of −0.4 for runoff comparison without calibration; after calibration, the *NSE* was 0.84 and *RE* was zero, and the *NSE* was 0.83 and *RE* was 0.32 during validation period. A *NSE* of 0.89 and *RE* of −0.16 for runoff were reported by Zema *et al*. [23] on the Ganspoel watershed (Belgium) without calibration. A NSE of 0.82 was achieved by Baginska *et al*. [24] on the Currency Creek experimental catchment of the Sydney Region (Australia) after calibration. Based on a thorough review of model evaluation methods done by Moriasi *et al*. [25], model simulation can be judged as satisfactory if *NSE* is greater than 0.50 and very good if *NSE* is greater than 0.75 for runoff. Thus, the overall model performance was good. Generally, the monthly runoff events were slightly underpredicted by AnnAGNPS, although a few monthly runoffs were over-predicted (Figure 2). As pointed out by Yuan *et al*. [12], and also demonstrated by Baginska *et al*. [24], Licciardello *et al*. [22] and Yuan *et al*. [14], AnnAGNPS tended to under-predict runoff due to the assumption of a triangular hydrograph and some approximations of model parameters. 

Annual average sediment yields monitored at the Wuxi station during 2000–2002 and 2003–2004 were 1.44 × 10^6^ t·y^−1^ and 1.09 × 10^6^ t·y^−1^, respectively [26]. Therefore, average sediment transport per square kilometer was calculated as 722 t·km^−2^·y^−1^ for the period of 2000-2002 and 546 t·km^−2^·y^−1^ for the period of 2003–2004, with the contribution area of 2000 km^2^ above the Wuxi station. Annual average sediment yields predicted by AnnAGNPS were 0.95 × 10^6^ t·y^−1^ for the period of 2000-2002 and 1.29 × 10^6^ t·y^−1^ for the period of 2003-2004 with the sediment transport rate of 477 t·km^−2^·y^−1^ and 645 t·km^−2^·y^−1^respectively, which are approximately 34% lower than the observed value during 2000–2002 and 18% higher than the observed value during 2003–2004. 

The use of RUSLE technology and the parameters associated with determining soil loss in AnnAGNPS are meant to be used as long term estimates. Yuan *et al*. [12] showed that AnnAGNPS adequately predicted average annual sediment load without calibration. Studies performed by Licciardello *et al*. [22] had a *NSE* of 0.51 and *RE* of 0.53 for sediment yield at event scale without calibration. A *RE* of 0.59 was achieved by Shrestha *et al*. [19] on a watershed in the Siwalik Hills of Nepal. Although no detailed observed sediment data were available for comparisons of the sediment simulation in this study, the *RE* of −0.34 and 0.18 without calibration indicates a satisfactory performance of AnnAGNPS application in simulation sediment. 

### 2.2. Watershed Soil Erosion and Sediment Yield Simulation of Current Conditions

Based on terrain, soil, land use, and crop management information and eight-year climatic data from eleven stations in the watershed, results predicted by AnnAGNPS were used in evaluating characteristics of soil erosion, sediment and sediment delivery of the watershed.

Soil erosion from individual cells was highly spatially variable (Figure 3). The soil loss tolerance in earth-rock mountainous areas of southwest China is 500 t·km^−2^ per year by the Ministry of Water Resources of China. 

Only 48% of the area of the total watershed, total area of 1,996.8 km^2^, is less than the tolerance value. The annual average soil erosion for the entire watershed is 1,551.7 t·km^−2^·y^−1^ (Table 2), which is much larger than the tolerance value. A small portion (8%) of the watershed had soil erosion in excess of 5,000 t·km^−2^·y^−1^ as shown in Table 2. Therefore, additional soil erosion control measures must be implemented to control soil erosion.

**Table 2:** Grades of soil erosion intensity predicted by AnnAGNPS for the watershed.

Grades	Soil erosion rate (t·km^−2^·y^−1^)	Area (km^2^)	Percent of area (%)	Average soil erosion rate (t·km^−2^·y^−1^)
Tiny	<500	1996.8	47.76	1,551.7
Slight	500–2,500	1348.6	32.26	
Middle	2,500–5,000	497.0	11.89	
Intense	5,000–8,000	238.5	5.70	
Extreme	8,000–15,000	99.2	2.37	
Violent	>15,000	0.8	0.02	

The soil erosion amount varied greatly with different land use types (Table 3). The forestland, shrub forestland and higher coverage grassland had low erosion amounts. The sloping land and lower coverage grassland covered 22% and 5% of the total watershed area, respectively, but their erosion amount accounted for 56% and 11% of the watershed total. Table 3 indicates that sloping land and lower coverage grassland are the main source of soil loss. Thus, the key of soil loss control is to control erosion from those lands and utilize those lands more rationally.

**Table 3:** Soil erosion of different land use types predicted by AnnAGNPS.

Land use types	Percent of area (%)	Soil erosion (×10^4^ t·y^−1^)	Percent of soil erosion (%)	Soil erosion rate (t·km^−2^·y^−1^)
Paddy field	0.36	0.13	0.02	86.7
Dry field	22.33	364.12	55.59	3900.1
Forestland	14.71	19.73	3.07	320.7
Shrub Forestland	45.28	111.15	17.32	587.1
Sparse forestland	1.56	9.33	1.52	1,430.9
Higher coverage grassland	1.26	1.92	0.38	364.4
Medium coverage Grassland	9.13	69.99	11.23	1,833.5
Lower coverage Grassland	5.34	72.43	10.90	3,242.3
Stream	0.02	0	0	0
Residential areas	0.01	0	0	0
Total	100.00	648.80	100.00	1,551.7

Soil erosion is the first step in the sedimentation processes which consist of erosion, transportation and deposition of sediments. A fraction of the eroded soil passes through the channel system and contributes to sediment yield, while much of soil deposits in landscape and water channels before reaching the outlet. Defining a sediment budget can help to better understand the sources, pathways and sinks (deposits) of sediment within a watershed system. The sediment budget of the watershed (Figure 4) shows that the average annual erosion amount of the Daning River watershed was 6.49 million tons, 21% deposited in the cells, 49% stored in the stream channels, and the rest was the output of the watershed. To reduce sediment loss at the watershed outlet, it is important to have more soil deposited in the cells. Thus, more conservation practices such as buffers should be adopted at the landscape scale to reduce sediment loss.

Table 4 shows annual average runoff and sediment characteristics from the main stem and six important tributaries in the watershed, which were also discretized by AGNPS 2001 data preparation tools using a CSA value of 8,000 ha and an MSCL value of 2,000 m. The contribution areas above Wuxi and Dachang stations are 2,000 and 3,050 km^2^, respectively. 

Among the six tributaries, Dongxi River, Xixi River, Houxi River, Boyang River, Bayanzhi River and Pingding River which altogether are about 73% of the entire watershed, Xixi River and Boyang River are the largest and smallest subwatersheds respectively (Table 4). 

The entire watershed can be classified into upper (above the Wuxing station), middle (between Wuxi and Dachang station) and lower (from Dachang station to the entire watershed outlet) watershed. Average annual sediment yield is 1.25 × 10^6^ t·y^−1^ at the Wuxing station of the upper watershed, rapidly adds up to 1.81 × 10^6^ t·y^−1^ at the Dachang station at the middle watershed, whereas it increases slowly in the lower watershed, with only 1.95 × 10^6^ t·y^−1^ at the watershed outlet. This indicates that the total 1.95 × 10^6^ t·y^−1^ sediment yield, 64% comes from the upper, 29% from the middle and 7% from the downstream watershed, respectively. Among the six tributaries, the Dongxi River, with the average annual sediment yield of 5.23 × 10^5^ t, about 27% of the total sediment yield, had the largest sediment contribution to the entire watershed. Dongxi River watershed, with a large amount of deep sloping lands and lower coverage grassland, will need more conservation measures implemented to reduce sediment loss. In addition, strategies of controlling bank erosion should also be sought to reduce sediment loss from this mechanism. Boyang River with the largest runoff, 565.2 mm·y^−1^ in six tributaries, yielded 3.16 × 10^5^ tons sediment per year, which was about 16% of the entire watershed. Bayangzhi River watershed had the least soil erosion of 766.6 t·km^−2^·y^−1^, followed by Pingding River watershed (1,054.9 t·km^−2^·y^−1^). The reason that these two subwatersheds had low soil erosion is that most of the watershed area was covered by forestland and shrub forestland. 

**Table 4:** Annual average runoff and sediment characteristics from main stem and six important tributaries predicted by AnnAGNPS in the Daning River watershed.

Name ^a^	Area (km^2^)	Runoff volume (×10^8^ m^3^·y^−1^)	PR ^b^(%)	Runoff depth (mm·y^−1^)	Sediment yield (×10^4^ t·y^−1^)	PSY ^c^(%)	STR ^d^ (t·km^−2^·y^−1^)	Soil erosion (×10^4^ t·y^−1^)	SER ^e^ (t·km^−2^·y^−1^)	PSE ^f^(%)
Dongxi River	542.0	2.7	14.52	501.8	52.3	26.88	965.8	114.3	2108.0	17.61
Xixi River	750.9	3.9	20.97	524.1	48.8	25.08	650.1	91.9	1223.9	14.17
Houxi River	514.8	2.8	15.05	540.7	24.0	12.33	465.8	63.7	1237.7	9.82
Boyang River	284.3	1.6	8.60	565.2	31.6	16.24	1,111.2	77.2	2715.0	11.90
BayanzhiRiver	463.0	1.4	7.53	299.9	19.0	9.76	410.3	35.5	766.6	5.47
Pingding River	485.1	1.8	9.68	378.1	19.5	10.02	402.8	51.2	1,054.9	7.89
Wuxi station	2,000.0	10.3	55.38	513.4	124.6	64.03	623.1	283.5	1,417.4	43.69
Dachang station	3,050.0	15.3	82.26	500.1	181.2	93.11	594.1	516.0	1,691.7	79.53
Watershed outlet	4,181.0	18.6	100.00	444.9	194.6	100.00	465.3	648.8	1,551.7	100.00

^a^ Name of subwatersheds and hydrological control stations; ^b^ Percent of the total runoff; ^c^ Percent of the total soil yield; ^d^ Sediment transport rate; ^e^ Sediment erosion rate; ^f^ Percent of the total soil erosion.

### 2.3. Evaluation of Sediment Delivery Ratio of the Watershed

Sediment yield is a critical factor in identifying non-point source pollution as well as in design and construction of dams and reservoirs. However, sediment yield is usually not available as a direct measurement but estimated by using a sediment delivery ratio (SDR) [27]. An accurate prediction of SDR is important in controlling sediments for sustainable natural resources development and environmental protection. 

Figure 5 shows the spatial distribution of SDR of the watershed. Estimated SDRs range from 0.376 to 0.531 in six tributaries, which is consistent with the ranges from 0.15 to 0.61 for main watersheds in the Three-Gorge area of the Yangtze River [28]. SDR of the whole watershed is approximately 0.30. The maximum SDR is 0.531 in the Xixi River watershed, and the minimum SDR is 0.376 in the Houxi River watershed. The reason for the variation of SDR is that each subwatershed varies greatly not only in the drainage area, but also in slope, relief-length ratio, runoff-rainfall factors, land use/land cover and sediment particle size. This SDR information is useful for future conservation planning of the watershed for effective soil erosion control and sediment loss.

## 3. Methods and Procedures

### 3.1. AnnAGNPS Model Description

The Annualized Agricultural Nonpoint Source Pollution (AnnAGNPS) model is an advanced technological watershed evaluation tool that has been developed through a partnership between the U.S. Department of Agriculture (USDA)—Agriculture Research Service (ARS) and the USDA—Natural Resources Conservation Service (NRCS) to aid in the evaluation of watershed responses to agricultural management practices [3,5,12,29]. AnnAGNPS is a continuous-simulation, daily time-step, pollutant loading model designed to simulate long-term chemical and sediment movement from agricultural watersheds; and it includes significantly more advanced features than AGNPS [6]. The spatial variability of soils, land use, and topography within a watershed is accounted for by dividing the watershed into many user-specified, homogeneous, drainage-area-determined cells. Runoff, sediment, and chemicals are routed from each cell through a channel network to the outlet of the watershed. The model has the capability to identify the sources of pollutants at their origin and to track them as they move through the watershed system. The surface runoff from a field is determined using the Soil Conservation Service Curve Number (SCS-CN) technique [30] within AnnAGNPS. The peak flow is calculated by the extended TR-55 technique [31]. The lateral subsurface flow and tile drainage are also accounted for in the model. Runoff in channels is calculated using Manning’s equation. Soil erosion from each field is predicted by Revised Universal Soil Loss Equation (RUSLE) [32]. The sediment yield leaving each field is based upon the Hydro–Geomorphic Universal Soil Loss Equation (HUSLE) [33]. The sediment reach routing is based on a modified Einstein deposition equation [34] using the Bagnold suspended sediment formula for the transport capacity by particle size class. The model can be used to study the effects of alternative cropping and tillage systems including the effects of fertilizer, pesticide, and irrigation application rates as well as point source yields and feedlot management [12].

Required input parameters for application of the model include climate data, watershed physical information, and management information. Daily climate information, which includes daily precipitation, maximum and minimum temperatures, dew point temperature, cloud cover, and wind speed, is needed to account for temporal variation in the weather. Climate data for simulation can be historically measured, synthetically generated using the climate generator program [35], or a combination of the two. GIS data layers of a watershed are extremely helpful in characterizing the watershed physical information. 

Using the GIS digital data layers of digital elevation model, soils, and land-use, a majority of the data input requirements of AnnAGNPS were developed by using a customized ArcView GIS interface. Inputs developed from the ArcView GIS interface include physical information of the watershed and subwatershed (AnnAGNPS cell), such as boundary and size, land slope and slope direction, and channel reach (AnnAGNPS reach) descriptions. The ArcView GIS interface also assigned a soil and land-use type to each cell by using the generated subwatershed and the soil and land-use GIS data layers. Additional steps to provide the model with the necessary inputs included developing the soil layer attributes to supplement the soil spatial layer, establishing the different crop operation and management data, and providing channel hydraulic characteristics. Those inputs can be organized using the AnnAGNPS Input Editor, a graphical user interface designed to aid users in selecting appropriate input parameters. Management information includes various field management operations such as planting, cultivation, fertilization, pesticides and harvesting, much of which can be obtained from RUSLE [31] databases or from actual activities implemented. 

Output information produced by the model includes runoff, sediment, nutrient and pesticide loads at a temporal scale ranging from daily to yearly and at any desired location such as specific cells, stream reaches, feedlots, gullies, or point sources. The model also has the capability to provide source accounting information in terms of the fraction of a pollutant load passing through any reach location that originated from an upstream watershed pollutant source area. Further details on the theoretical background of AnnAGNPS can be found in the literature [3,5,30,36]. 

### 3.2. Watershed Description

**Figure 6:**
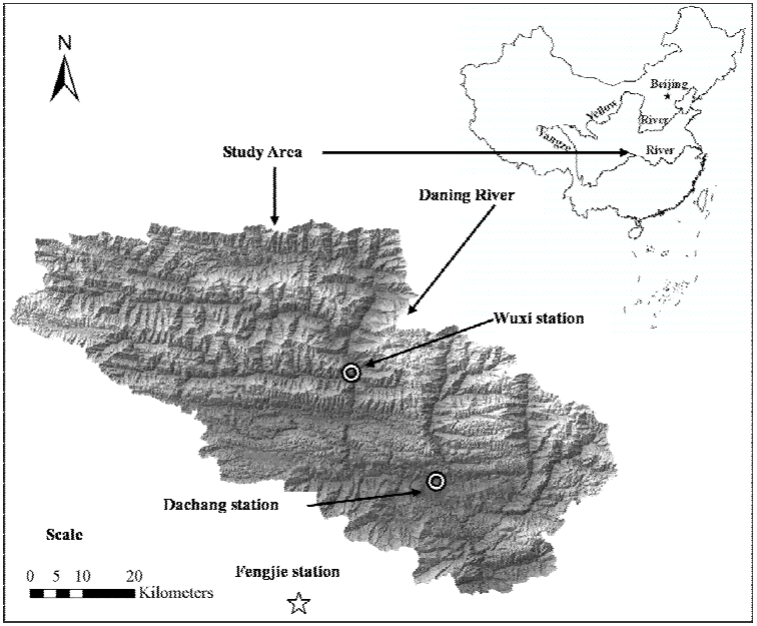
Location of the study watershed.

The study area is the Daning River watershed (108°44' to 110°11' E, 31°04' to 31°44' N), which is located in the center of the Three-Gorge reservoir area of the Yangtze River, China (Figure 6). The entire watershed has a contributing drainage area of about 4,181 km^2^ and drains into the Yangtze River. The terrain is undulating, ranging from 95 m to 2,793 m above sea-level, and the slope varies from 0 to 138%, which is derived from 1:50,000 DEM. The region has a subtropical humid-warm climate, with annual average temperatures of around 19.8 °C. Annual rainfall ranges from 1,000 mm in the valleys to 1,700 mm in the high mountain areas of the watershed with large variation from year to year. Over 87% of the rainfall occurs between April and October, which markedly affects runoff and soil erosion. The main crops grown in the Daning River watershed are rice, wheat, maize and rape. The soils in the study area vary from sandy loams to loamy sands with most of the soil types in the watershed classified as clay loam. The dominant soil in the watershed is yellow soil and yellow limestone soil, which represents almost 50% of the watershed. Major soil types in the watershed and their basic physical and chemical properties are listed in Table 5. 

**Table 5:** Predominant soils of the Daning River watershed and their basic characteristics (Chinese Soil Taxonomic Classification).

Soil	Area (%)	HG ^a^	Clay (%)	Silt (%)	Sand (%)	Rock (%)	pH	OM^ b^(%)	*K* (t·ha·h·ha^−1^·MJ^−1^·mm^−1^) ^c^	Field capacity ^d^	Wilting point ^d^	Saturated water conductivity (mm·h^−1^)
										(cm^3^water· cm^−3^soil)		
Yellow soil	34.6	D	26.5	37.7	35. 8	13.7	5.9	2.9	0.033	0.291	0.152	4.9
Skeletal yellow soil	10.0	B	14.0	41.2	44.7	16.3	7.9	1.5	0.040	0.242	0.104	16.5
Yellow-brown soil	18.6	D	30.1	34.1	35.9	26.3	5.8	3.6	0.030	0.305	0.169	3.6
Skeletal yellow-brown soil	6.3	D	29.9	37.6	32.4	13.7	6.1	2.5	0.034	0.310	0.168	3.9
Brown soil	7.3	B	21.6	41.5	36.8	21.4	5.5	7.6	0.033	0.273	0.131	7.7
Yellow limestone soil	14.9	D	28.5	34.6	36.9	42.8	5.5	2.7	0.032	0.297	0.162	4.0
Primary calcareous purple soil	4.2	B	22.6	39.9	37.5	2.2	6.7	3.5	0.032	0.275	0.135	6.9
Mountain meadow soil	1.0	B	20.1	38.1	41. 8	11.8	5.4	10.0	0.031	0.260	0.126	8.4
Other	3.1											

^a^ Soil Hydrological group; ^b^ Organic matter; ^c^ Soil erodibility factor; ^d^ Derived from Saxton *et al*.(1986) based on clay and sand ratios.

Detailed records of agricultural operations including tillage, planting, harvesting, and fertilization have been maintained since 1998. The operation management of four main crops related to this study is listed in Table 6.

**Table 6:** Major crops grown, management schedules and management operations identified in the watershed.

Management schedules	Event date (day/month)	Management operations	Crop
Rice-Rape	10/05	Tillage/Fertilizer	Rice
	11/05	Irrigation	
	18/05	Fertilizer	
	3/06	Fertilizer	
	5/07	Fertilizer	
	10/9	Harvesting	
	11/10	Tillage/Fertilizer	Rape
	15/10	Fertilizer	
	15/10	Seeding	
	10/11	Fertilizer	
	26/02	Fertilizer	
	5/05	Harvesting	
Maize-wheat	23/05	Tillage/Fertilizer	Maize
	26/05	Fertilizer	
	26/05	Seeding	
	6/07	Fertilizer	
	13/09	Harvesting	
	2/11	Tillage/Fertilizer	Wheat
	5/11	Fertilizer	
	5/11	Seeding	
	12/02	Fertilizer	
	10/03	Fertilizer	
	15/05	Harvesting	

Daily runoff data at the Wuxi monitoring station during 1998 and 2005 were obtained from the Ministry of Water Resources of China. Runoff was monitored using a velocity instrument method, *i.e.*, the two point method and the eleven point method. The two point method, employed for the low water level in the station, is that velocities are sampled at two tenths and eight tenths of the water depth, and the results are averaged. The eleven point method, used for the high water level, is the same as the two point method, but velocities are sampled at eleven points of the water depth (top, one tenths, two tenths, three tenths, four tenths, five tenths, six tenths, seven tenths, eight tenths, nine tenths and bottom). Unfortunately, the data between 2000 and 2002 were missing. In addition, detailed sediment data were also unavailable.

### 3.3. Input Preparation

#### 3.3.1. Climate Data

**Figure 7:**
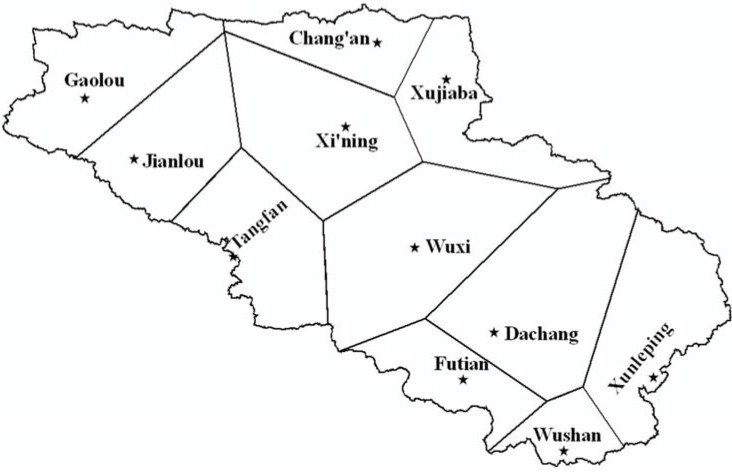
Spatial distribution of raingauges and associated Thiessen polygons in the Daning River watershed.

Required data for the weather file for model application includes daily precipitation, maximum and minimum temperatures, dew point temperature, sky cover and wind speed. There are a total of 11 rain gauges in the Daning River watershed. Due to large spatial and temporal variations of the precipitation in the watershed, multiple weather files were used. The entire watershed was divided into 11 polygons using the Thiessen polygon method [37]. Each polygon contains one rainfall station and any AnnAGNPS cells within the same polygon have the same rainfall data. The weather file for each polygon was created using recorded precipitation data from the rain gauges in the polygon for the time period of January 1998 to December 2005 (Figure 7). Other weather information for the same period was available from Fengjie meteorological station approximately 40 km away from Wuxi station, which is the nearest climate station for the watershed. 

#### 3.3.2. Topographic Data

**Figure 8:**
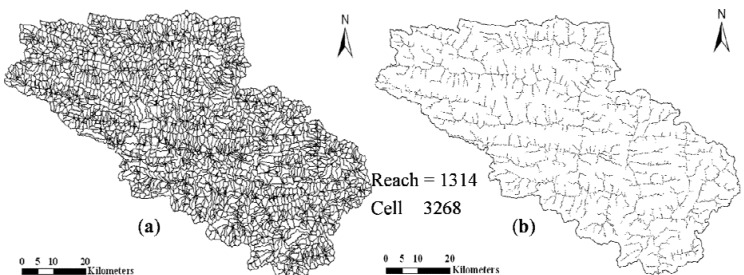
Generated subwatersheds and stream network of the study watershed.

Topographic parameterization software, the AGNPS 2001 data preparation tool, was used for digital landscape analysis of 25-m raster Digital Elevation Model (DEM). The size of the cells was determined by a user-defined critical source area (CAS) of 150 ha and a minimum source channel length (MSCL) of 100 m. As a result of processing the DEM data, the study area was discretized into 3,268 drainage areas (amorphous cells) and 1,314 reaches (Figure 8). The watershed and associated sub-watershed boundaries (Figure 8a) were delineated and stream network (Figure 8b) was generated. Terrain-based geomorphic and drainage parameters containing cell area, slope, perimeter, RUSLE LS-factor, channel segment length and slopes, and the topology of the cell network were also calculated.

#### 3.3.3. Land Use

The land use map was developed from Landsat 5 TM satellite image of October, 2004. Five major types of land use: farmland (paddy field and dry field), forest (forestland, shrub forestland, sparse forestland), grassland (higher, medium and lower coverage grassland), stream and residential areas, were identified in the watershed. The dominant land use was assigned to each AnnAGNPS cell.

#### 3.3.4. Crops and Cultivation Practices

Crop management operation information reflecting the effect of human activities on the watershed is important to determine the sediment yield accurately. Therefore, the operation management information should be developed with as much detail as possible; especially for those operations that cause soil disturbance or land cover changes. Crop operation and field management data in the watershed were prepared based on field investigation, RUSLE guidelines and databases. The predominant crops grown were maize, rice, rape and wheat. The major crop rotation patterns are rice-rape in paddy fields and maize-wheat in dry (upland) land (Table 6).

#### 3.3.5. Soil Data

In the model, 12 parameters from a total of 28 soil-based parameters were required for runoff and erosion simulation. Required inputs included particle size fraction, bulk density, albedo, saturated hydraulic conductivity, field capacity, and wilting point. The dominant soil type was determined for each AnnAGNPS cell, and associated characteristics for that soil type were organized through the Input Editor. Soil data mainly came from the Soil Survey Office in Sichuan Province of China [38]. However, some necessary soil information for AnnAGNPS simulation was not available. Using the “Soil water characteristics” software [39], the soil hydraulic parameters such as saturated hydraulic conductivity, field capacity and wilting point ratios were derived. The proportion of very fine sand (VFS) in the soil was estimated as the product of sand and silt divided by 100 [19]. The soil erodibility factor was estimated using the equation developed by Sharply *et al*. [40].

#### 3.3.6. SCS Curve Number

The SCS-CN is a key factor to obtain accurate prediction of runoff and sediment yields. Curve numbers were selected based on the National Engineering Handbook, Section 4 [41] with some adjustment to incorporate local conditions. Adjustment was made during the model calibration processes, and was done by trial and error using the graphical comparison as well as the comparison of statistical parameters of observed and predicted runoff. The estimated SCS-CN values for different land uses of the watershed were listed in Table 7. The CN for four crops, *i.e.*, wheat, maize, rice and rape was used when the crops were growing. And the CN for fallow with residue was used when one crop was harvested but another crop had not yet been planted. Further, the CN for other land use types were selected and listed in Table 7 too.

**Table 7:** Estimated SCS curve numbers for the Daning River watershed used in the model simulations.

Curve number Hydrologic soil group				
Land cover class	A	B	C	D
Corn straight row (Poor)	65	75	82	86
Rice straight row (Poor)	63	74	82	85
Wheat straight row (Poor)	63	74	82	85
Rape straight row (Poor)	64	75	83	85
Lower coverage Grassland	50	80	87	93
Medium coverage Grassland	40	71	81	89
Higher coverage grassland	30	62	74	85
Forestland	30	55	70	77
Shrub forestland	36	60	73	79
Sparse forestland	45	66	77	83
Urban	89	92	94	95
Fallow + Crop residue (Poor)	76	85	90	93

### 3.4. Model Evaluation Criterion

Runoff data from 1998–1999 were used for calibration and data from 2003–2005 were used for validation. The purpose of calibration is to achieve a satisfactory simulation through an iterative procedure of parameter evaluation and refinement, as a result of comparing simulated and observed values of interest. The purpose of the model validation is to determine the quality of the model predictions for other time periods not considered in calibration. The following statistics were used to help evaluate model performance for calibration: the mean and standard deviation of both observed and simulated values, the relative error (*RE*), the gradient k, intercept b, and coefficient of determination (*R*^2^) of linear regression, the Nash-Sutcliffe coefficient of efficiency (*NSE*) [42], and root mean square error (*RMSE*).

The RE is the ratio of the total difference between simulated and observed values *versus* the total observed value. It ranges from minus one to ∞ while zero indicates that there is no difference between model simulation and field observation. The smaller the absolute value of a RE, the better performance of the model is. 

The coefficient of determination, *R*^2^, gives the proportion of the variance in the observed values explained by the simulated values. For a good agreement the gradient k should be close to one and the intercept b should be close to zero. 

The NSE is a technique often employed to evaluate simulated results in hydrological modeling. It is calculated according to Equation (1):


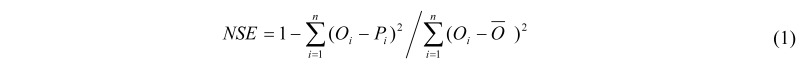


where *O*_i_, *P*_i_ and *ō* are observed, predicted, and the mean of the observed value respectively; and *n* is the total number of events. The NSE ranges from −∞ to 1 while one indicates that the model is perfect. A value larger than zero indicates that the model is minimally acceptable. Values between zero and one indicate that the model is a better indicator than the mean of the observed values; and negative values indicate that the mean of the observed values is a better indicator than the model [42]. 

The RMSE describes the difference between the observed and simulated values in the unit of the variable [23,43]. The RMSE range from 0 to ∞ while zero indicates there is no difference between model simulation and field observation. The RMSE can be expressed by Equation (2):


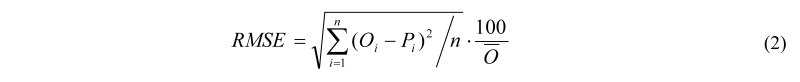


AnnAGNPS did not simulate base-flow, hence to compare the model predicted runoff to observed runoff, baseflow was separated from the observed runoff using the baseflow filter method [44].

### 3.5. Sediment Delivery Ratio (SDR)

SDR, a sediment transmission coefficient, is calculated as the ratio of sediment yield at the watershed outlet (point of interest) to the gross erosion in the entire watershed. It can be obtained from the following Equation:


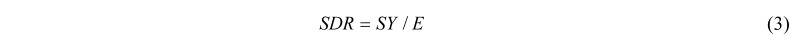


where SDR is the sediment delivery ratio. SY is the sediment yield, and E is the gross erosion per unit area above a measuring point. 

## 4. Conclusions

AnnAGNPS was used in predicting runoff and sediment yield for a Three-Gorge watershed of the Yangtze River, China. The study demonstrates that AnnAGNPS adequately predicts long-term monthly runoff with *RE* = 0.01, *NSE* = 0.94 and *R*^2^ = 0.94 (*p* < 0.05) in the calibration period; *RE* = −0.06, *NSE* = 0.93 and *R*^2^ = 0.93 (*p* < 0.05) in the validation period. The comparison of annual average sediment yield monitored during 2000–2002 and 2003–2004 in the Wuxi station also achieved satisfactory agreement with *RE* of −0.34 and 0.18 respectively. The predicted runoff and sediment yield compared well with the observed data indicating that the model has an acceptable performance in simulation of runoff and sediment yield for the Daning River watershed. Additional model simulation showed that soil erosion from individual cells was highly spatially variable. Approximately 48% of the watershed had 0–500 t·km^−2^·y^−1^. However, 8% of the area had soil erosion in excess of 5,000 t·km^−2^·y^−1^. Sloping lands and lower coverage grassland were the main source of soil loss in the watershed, indicating the key area of soil loss control. The average annual erosion of the Daning River watershed was 6.49 million tons, and of which about 21% was deposited in the cells and 49% was stored in the stream channels, with the rest (30%) delivered to the watershed outlet. This indicates that more erosion control should be sought on the landscape to reduce soil loss. The SDR of the entire watershed is 0.3 and it varies greatly for each subwatershed. The results obtained from applying AnnAGNPS on this Three-Gorge watershed demonstrate that the model has considerable potential as a research and management tool for comparative assessment, long-term monthly and annual estimation of runoff and sediment yields, identification of landscape “hot spots”, and exploration of sediment delivery characteristics. 
